# Efficacy and safety of enasidenib and azacitidine combination in patients with *IDH2* mutated acute myeloid leukemia and not eligible for intensive chemotherapy

**DOI:** 10.1038/s41408-021-00604-2

**Published:** 2022-01-25

**Authors:** Sangeetha Venugopal, Koichi Takahashi, Naval Daver, Abhishek Maiti, Gautam Borthakur, Sanam Loghavi, Nicholas. J. Short, Maro Ohanian, Lucia Masarova, Ghayas Issa, Xuemei Wang, Bueso-Ramos Carlos, Musa Yilmaz, Tapan Kadia, Michael Andreeff, Farhad Ravandi, Marina Konopleva, Hagop M. Kantarjian, Courtney D. DiNardo

**Affiliations:** 1 Department of Leukemia, The University of Texas MD Anderson Cancer Center, Houston, TX USA; 2 Department of Hematopathology, The University of Texas MD Anderson Cancer Center, Houston, TX USA; 3 Department of Biostatistics, The University of Texas MD Anderson Cancer Center, Houston, TX USA

**Keywords:** Acute myeloid leukaemia, Drug development

## Abstract

Preclinically, enasidenib and azacitidine (ENA + AZA) synergistically enhance cell differentiation, and venetoclax (VEN), a small molecule Bcl2 inhibitor (i) is particularly effective in *IDH*2 mutated acute myeloid leukemia (*IDH2*^*mut*^AML). This open label phase II trial enrolled patients (pts) with documented *IDH2*^*mut*^AML. All patients received AZA 75 mg/m^2^/d x 7 d/cycle and ENA 100 mg QD continuously. Concomitant Bcl2i and FLT3i were allowed (NCT03683433).Twenty-six pts received ENA + AZA (median 68 years, range, 24–88); 7 newly diagnosed (ND) and 19 relapsed/refractory (R/R). In R/R AML patients, three had received prior ENA and none had received prior VEN. The composite complete remission rate (CRc) [complete remission (CR) or complete remission with incomplete hematologic recovery (CRi)] was 100% in ND AML, and 58% in R/R AML. Median OS was not reached in ND AML with median follow-up of 13.1 months (mo); Pts treated in first relapse had improved OS than those with ≥2 relapse (median OS not reached vs 5.2 mo; HR 0.24, 95% CI 0.07–0.79, *p* = 0.04). Two patients received ENA + AZA with a concomitant FLT3i, one responding ND AML patient and one nonresponding R/R AML patient. Seven R/R AML pts received ENA + AZA + VEN triplet, and with median follow up of 11.2 mo, median OS was not reached and 6-mo OS was 70%. The most frequent treatment-emergent adverse events include febrile neutropenia (23%). Adverse events of special interest included all-grade IDH differentiation syndrome (8%) and indirect hyperbilirubinemia (35%). ENA + AZA was a well-tolerated, and effective therapy for elderly pts with *IDH2*^*mut*^ ND AML as well as pts with R/R AML. The addition of VEN to ENA + AZA appears to improve outcomes in R/R *IDH2*^*mut*^AML.

Clinical trial registration information: https://clinicaltrials.gov/.NCT03683433

## Introduction

Until recently, older patients with acute myeloid leukemia (AML) not eligible for intensive chemotherapy were treated with azacitidine (AZA), demonstrating response rates of 25–31% and a median overall survival (OS) under 12 months in newly diagnosed (ND) patients [1–4]. In November 2018, venetoclax (VEN), a small molecule Bcl2 inhibitor (i) in combination with hypomethylating agent (HMA: AZA or decitabine) was approved for the treatment of ND AML in patients aged ≥75 years and/or not eligible for intensive chemotherapy. ND AML patients treated with VEN + AZA demonstrated 66.4% composite complete remission rate (CRc) [complete remission (CR) or complete remission with incomplete hematologic recovery (CRi)] and a median OS of 14.7 months, with particularly favorable outcomes in those harboring isocitrate dehydrogenase 2 *(IDH2*) gene mutations [5]. Despite these marked strides in the treatment of AML, the majority of patients with AML still experience relapse, emphasizing the unmet need for effective therapies adept at inducing durable remission [6].

*IDH2* mutations (*IDH2*^mut^)occur in approximately 12–15% of patients with AML, and occur with increasing frequency in older patients [7]. Wild-type IDH2 catalyzes the conversion of isocitrate to α-ketoglutarate in the Krebs cycle; whereas, mutant IDH2 neomorphically generates the oncometabolite 2-hydroxyglutarate (2-HG), leading to DNA hypermethylation and myeloid cell differentiation arrest, promoting leukemogenesis [8]. Enasidenib (ENA), a first-in-class small molecule mutant IDH2i is approved as monotherapy in R/R *IDH2*^*mut*^AML [9]. In the R/R setting, ENA (*n* = 171) demonstrated an overall response rate [ORR, including CR, CRi, partial remission (PR), and morphologic leukemia-free state (MLFS)] of 40.3%, with 19.3% CR, and a median OS of 8.8 months [9]. In a smaller cohort of ND patients with *IDH2*^*mut*^ AML (*n* = 39) treated within the same study, ENA showed an ORR of 30.8%, including 18% CR, and a median OS of 11.3 months [10].

Preclinically, ENA + AZA combination synergistically promoted differentiation, and potentiated leukemic cell death [11]. Interim results of the ongoing randomized study evaluating ENA + AZA in patients with ND *IDH2*^*mut*^AML reported a favorable ORR of 71%, with 53% CR rates and a median OS of 22 months [12]. The effectiveness of this combination in the R/R *IDH2*^*mut*^ AML is unknown. Additionally, the Bcl2-inhibitor venetoclax (VEN) is particularly effective in *IDH2*^*mut*^AML as the accumulation of the oncometabolite 2-HG inhibits cytochrome C oxidase and effectively primes AML blasts to Bcl2 inhibition. Preclinically, ENA + VEN combination demonstrated improved survival in murine models, as compared to either agent alone or with sequential use of the two agents [13, 14]. Herein, we report our experience with the combination of ENA + AZA as frontline or salvage AML therapy (NCT03683433) for *IDH2*^*mut*^ AML, with or without the addition of VEN.

### Study design and participants

This single-center, phase 2 trial enrolled patients with *IDH2*^*mut*^ AML and [1] age ≥60 years with ND AML and ineligible for intensive chemotherapy [2], age >18 years with treated or untreated secondary AML arising from an antecedent hematologic neoplasm [15] and/or [3] R/R AML. Eligibility required an Eastern Cooperative Oncology Group (ECOG) Performance Status ≤3 and adequate end-organ reserve. Prior treatment with ENA, AZA, and/or VEN was allowed. The study was approved by the Institutional Review Board at the University of Texas, MD Anderson Cancer Center and conducted in accordance with the Declaration of Helsinki.

### Procedures

Patients received daily AZA (IV or SQ 75 mg/m^2^ x 7 days) and ENA 100 mg orally continuously per 28-day cycle. Cytoreduction with hydroxyurea and/or 1 dose of cytarabine (up to 2 g/m^2^) prior to treatment initiation was allowed. As *IDH2*^mut^ are known to frequently co-occur with activating *FLT3*^mut^ [7], approved FLT3i such as sorafenib, gilteritinib or midostaurin were allowed, as clinically appropriate [16]. Similarly, the addition of VEN in combination with AZA has been shown to be particularly effective in patients with *IDH2*^mut^ AML; therefore, the addition of VEN was allowed in combination with ENA and AZA, as clinically indicated [5, 17]. Patients remained on study until disease progression, unacceptable toxicity, or transition to allogeneic hematopoietic stem cell transplant (HSCT). All adverse events (AE) were monitored during the study protocol and categorized as per the Common Terminology Criteria for Adverse Events (CTCAE version 4.03)

### Outcomes

The primary outcome of this phase II trial was to assess the efficacy as measured by the composite CR rate (CRc = CR + CRi) within the first four cycles of therapy. Secondary outcomes included OS, Event-free survival (EFS), duration of response (DoR), and toxicity. OS was measured from the start of treatment until the date of death due to any cause or censored at the date of the last follow-up. EFS was measured from the date of treatment start until treatment failure, relapse, or death from any cause, whichever occurred first. Patients who were alive and without disease relapse at the time of the last follow-up were censored. Duration of response is defined, among responders, as the time duration between the date of response and the date of disease relapse or death from any cause, whichever occurred first. Patients who were alive and without disease relapse at the time of the last follow-up were censored. Survival endpoints were not censored at HSCT.

### Exploratory outcomes

Immunophenotypic evidence of measurable residual disease (MRD) was performed using eight-color flow cytometry. MRD was quantified as percentage of total leukocytes after the exclusion of most red blood cell precursors. At least 200,000 events were acquired to achieve a minimum sensitivity of 10^−3^–10^−4^ (0.1–0.01%), as described previously [18]. Next-generation sequencing (NGS) was employed for the assessment of mutations. The assay interrogates the entire exonic or hotspot regions of 81 genes that are frequently mutated in myeloid malignancies, as described previously [19]. A minimum sequencing coverage of x250 was used to reach an analytical sensitivity of 1% mutant reads in a background of wild-type. *FLT3* mutation analysis was performed using a multiplex fluorescent-based polymerase chain reaction (PCR) analysis followed by capillary electrophoresis.

Pre-specified exploratory objectives included an analysis of the relationship between co-occurring mutations, rate of *IDH2* mutation clearance by NGS, the incidence of MRD-negativity by flow cytometry, and the incidence and characteristics of IDHi-associated differentiation syndrome (IDH-DS) with combination therapy.

### Statistical Considerations

This study was not designed for comparison. We focused on the estimation of activity of the combination therapy, using the method of Thall, Simon, and Estey for futility and toxicity monitoring [20]. The trial was designed with a maximum sample size of 50. With this sample size, the half-width of the 95% confidence interval will be at most 0.14 when estimating the CRc rate. For the purpose of futility monitoring, our target CRc rate is 40% (desirable), while a CRc rate of 30% or lower will be considered not desirable. The Bayesian interim futility monitoring will be applied in cohort size of 10 and if at any interim look, we determine that there is less than 5% chance that the CRc rate improves over historical rate by more than 10%, the trial will be stopped early due to futility. Based on this stopping rule, the trial will be stopped early if we observe < /= 1 out of 10, 4 out of 20, 7 out of 30, or 11 out of 44 patients with CRc. Similarly, we will implement the Bayesian toxicity monitoring rule in cohort size of 10 and the trial will be stopped early if, at any interim look, there is more than 90% chance that the toxicity rate is more than 30%. No early futility or toxicity stopping rules were met and the study continues to enroll.

All patients who received at least one dose of the combination therapy were evaluable for safety and response assessments regardless of the duration of treatment. Patient characteristics were summarized using frequency (percentage) for categorical variables and median (range) for continuous variables. Toxicity type and severity were summarized. Time-to-event endpoints including OS, EFS, and DoR were estimated using the method of Kaplan-Meier and log-rank tests were used to compare between subgroups. All analyses were done using Prism, GraphPad (version 8.4).

## Results

### Patient characteristics

**Fig. 1:**
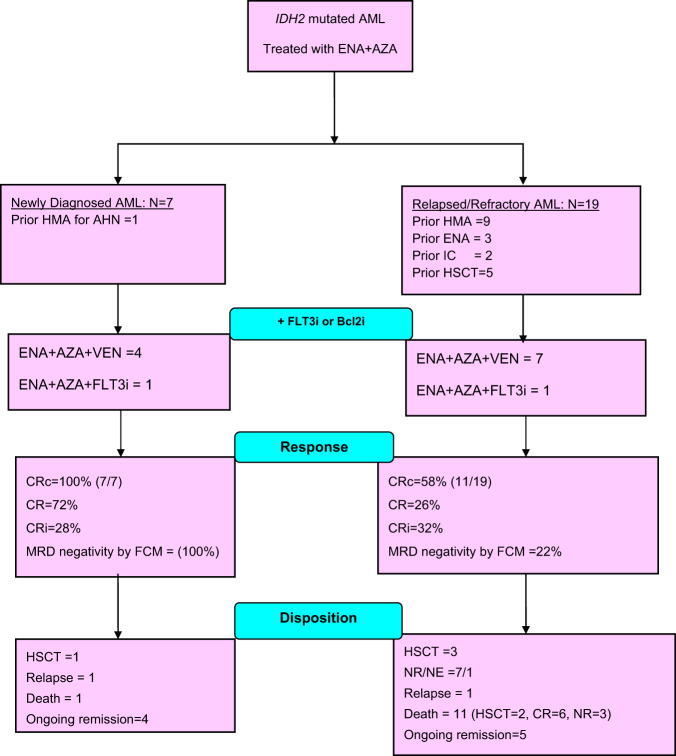
Consort diagram of Phase II trial of ENA+AZA combination in *IDH2*^mut^ AML. Consort diagram. AML- Acute myeloid leukemia, ENA- Enasidenib, AZA- Azacitidine, HMA- hypomethylating agent, AHN- antecedent hematological neoplasm, IC- intensive chemotherapy, HSCT- Hematopoietic stem cell transplant, i- inhibitor, VEN- venetoclax, CRc- composite complete remission, CR- complete remission, CRi- complete remission with incomplete hematological recovery, MRD- measurable residual disease, FCM -Flowcytometry, NR -no response, NE- not evaluable.

Twenty-six patients were enrolled between September 20, 2018 and October 7, 2020, (ND = 7, R/R = 19). The consort flowchart is shown in Fig. 1, and baseline characteristics in Table 1

**Table 1:** Baseline Demographics.

Baseline Characteristics	Total (*n* = 26)	Newly diagnosed *n* = 7	Relapsed/refractory *n* = 19
Age (years)	68 (24–88)	77 (66–81)	64 (24–88)
Sex (male)	20 (84)	6 (86)	14 (82)
**Performance status**			
ECOG 0	1 (4)	1 (14)	0
ECOG 1	18 (69)	4 (57)	14 (74)
ECOG ≥ 2	7 (28)	2 (29)	5 (26)
**Hematological parameters**			
Hemoglobin (mg/dL)	8.6 (6.2–13.7)	8.4 (6.2–10.6)	8.8 (7.1–13.7)
White Blood Count, x10^9^/L	3.8 (0.4–270)	6 (2–270)	1.5 (0.4–36)
Platelet, x10^9^/L	52 (12–1145)	74 (30–1145)	30 (12–223)
Circulating blasts (%)	20 (0–95)	34 (10–95)	5 (0–66)
Bone marrow blasts (%),	39 [4–87]	57 [20–86]	20 [4–87]
**ELN 2017 risk group**			
Intermediate	14 (59)	5 (71)	9 (47)
Adverse	12 (41)	2 (29)	10 (53)
**Diagnosis**			
In first relapse		0	8(42)
In second or later relapse		0	11 (58)
**Prior AML therapies**			
HMA	9 (28)	1 (14)	9 (42)
ENA	3 (12)		3 (16)
HSCT	5 (19)		5 (26)
**Co-occurring mutations**			
*ASXL1*	5 (19)	2 (29)	3 (16)
*DNMT3A*	8 (31)	2 (29)	6 (31)
*FLT3*	3 (12)	2 (29)	1 (5)
*NPM1*	5 (19)	1 (14)	4 (21)
*(K/N) RAS*	3 (12)	0	3 (16)
*RUNX1*	6 (23)	2 (29)	4 (21)
*SRSF2*	10 (38)	5 (71)	5 (26)
*TET2*	5 (19)	1 (14)	4 (21)
*TP53*	2 (8)	0	2 (11)

*ECOG-* Eastern Cooperative Oncology Group, *ELN-* European Leukemia Net, *HMA-* Hypomethylating agent, *ENA-* Enasidenib, *HSCT-* Hematopoietic cell transplantation.

All results expressed as No. (%) or median [Minimum–maximum], unless specified.

Among patients with ND *IDH2*^mut^AML (*n* = 7), the median age was 77 years (range, 66–81); one patient had prior HMA exposure for an antecedent hematological neoplasm. Among those with R/R *IDH2*^mut^ AML (*n* = 19), the median age was 64 years (range, 24–88). Eight patients were in first relapse (42%) and eleven (58%) in the second relapse or beyond. Patients with R/R AML had received a median of two prior therapies (range,1–4) of which nine (47%) had prior HMA and three (16%) had prior ENA exposure, two patients had received prior intensive chemotherapy (11%) and five (26%) had relapsed post-HSCT. None had prior VEN exposure.

Among patients with ND *IDH2*^mut^AML, 5 (71%) were categorized as intermediate risk and two patients (29%) with adverse risk according to ELN 2017 [21]; all seven (100%) patients had AML with *IDH2* p.R140Q with a median variant allele frequency (VAF) of 42% (range, 26–50%). This cohort was enriched in co-occurring mutations in *SRSF2* (71%, *n* = 5), and *FLT3* (29%, *n* = 2 one internal tandem duplication (ITD) and one tyrosine kinase domain point mutation (TKD); no concurrent *TP53*^mut^ were detected. Contrastingly in R/R *IDH2*^mut^AML, ELN adverse risk was more frequent (53%) compared with intermediate risk (47%). Among patients with R/R AML, 14 (74%) harbored *IDH2* p.R140Q and 5 (26%) had *IDH2* p.R172K mutations with median VAFs of 23% (range, 3–50%), and 35% (range, 18–46%), respectively. The R/R cohort was notable for concurrent mutations involving *RUNX1* (21%), *KRAS/NRAS* (16%), *TP53* (11%), and *FLT3* -ITD (5%).

### Efficacy

Twenty-five patients were evaluable for response, summarized in Table 2. Among patients with ND *IDH2*^mut^ AML, the CRc (CR/CRi) was 100% (*n* = 7) with 72% (*n* = 5) achieving a CR. Among the two patients who achieved CRi, one patient (50%) achieved hematological improvement in both hemoglobin and platelets. All seven patients (100%) additionally achieved MRD negativity by flow cytometry (MRD^neg^ FCM). Four patients received concurrent venetoclax (ENA + AZA + VEN), and one patient with comutated *FLT3*-ITD received concurrent gilteritinib. One patient (14%) underwent HSCT in MRD^neg^ FCM remission; notably, this patient had prior HMA exposure and had received the “triplet” of ENA + AZA + VEN. Among the responders, the median duration of response was not reached (range, 1.6-not reached) in the entire cohort and in the four patients receiving the ENA + AZA + VEN triplet and was 7.5 months in the one patient receiving the ENA + AZA + gilteritinib triplet. At a median follow-up of 13.1 months, median OS and EFS were not reached, and the 1-year OS and EFS rates were 83% and 56%, respectively.

**Table 2:** Outcomes in newly diagnosed and relapsed/refractory patients with *IDH2* mutant acute myeloid leukemia.

Response	Newly diagnosed *n* = 7	Relapsed/refractory *n* = 19
CRc	7 (100)	11 (58)
CR	5 (72)	5 (26)
CRi	2 (28)	6 (32)
MRD negativity by FCM	7/7 (100)	2/9 (22)
Not evaluable	0	1 (5)
No response	0	7 (37)
Median number of cycles given (range)	3 (1–8)	4 (1–17)
Median time to best response, months (range)	1.6 (1.0–4.2)	1.8 (0.8–5.4)

*CRc* -composite complete remission rate = CR + CRi, *CR-* complete remission, *CRi* -CR with incomplete hematologic recovery, *MRD-* measurable residual disease, *FCM-* flowcytometry

All results expressed as No. (%) or median [Minimum–maximum], unless specified.

Among 19 patients with R/R *IDH2*^mut^ AML, 18 were evaluable for response, the CRc was 61% (*n* = 11) with 28% (*n* = 5) achieving a CR. Hematological improvement was not noted in this heavily pretreated cohort. The MRD^neg^ FCM rate was 22% (*n* = 2/9) among the responding patients; notably both of them received ENA + AZA + VEN triplet combination. Seven patients were refractory to treatment including a patient with *FLT3*-ITD co-mutation with relapsed AML post-HSCT and received ENA + AZA + midostaurin, and another pt who received ENA + AZA + VEN triplet combination. Among the eleven responders, six patients received ENA + AZA + VEN, among which five had prior HMA exposure, and one had prior ENA exposure. The CRc in patients treated with ENA + AZA + VEN was 86% (n = 6/7) with a CR rate of 71% (n = 5/7). Among patients with ECOG ≥ 2, the CRc rate was 80% (*n* = 4/5), and among those with prior HMA, ENA and HSCT exposure, the CRc rates were 67% (*n* = 6/9), 100% (*n* = 3/3), and 60% (*n* = 3/5) respectively. Patients in first relapse had numerically higher CRc (75%, *n* = 6/8) than those in second relapse or higher (CRc: 50%, *n* = 5/10). Three patients (17%) underwent HSCT, among which two were in second relapse or higher.

**Fig. 2:**
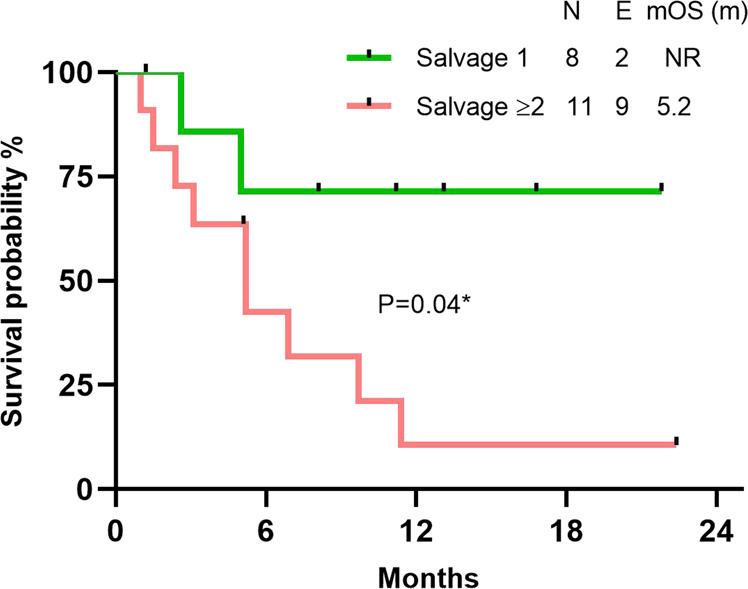
Kaplan–Meier plot showing overall survival (OS) by line of therapy in R/R cohort. N number, E events,mOS (m) median overall survival in months.

**Fig. 3:**
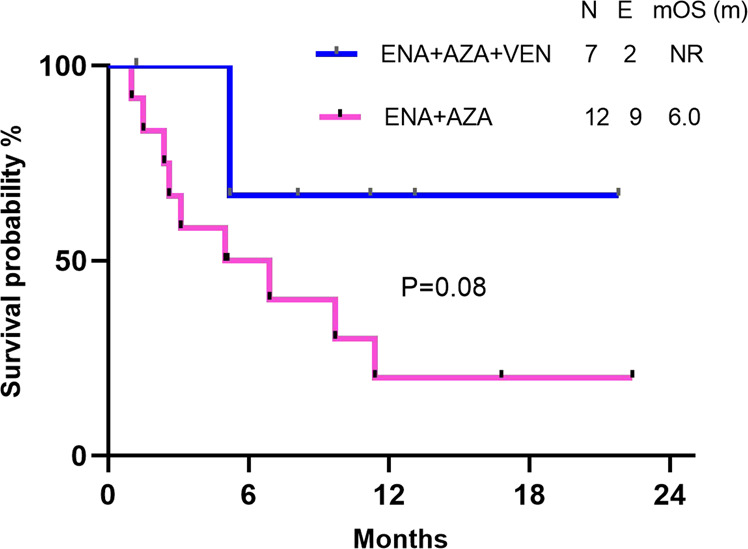
Kaplan–Meier plot showing overall survival (OS) by venetoclax status in R/R cohort. N number, E events,mOS (m) median overall survival in months.

Among the responders, the median duration of response was not reached. At a median follow up of 13.1 months, median EFS was 6.9 months with a 1-year EFS rate of 25%. Among the responders in the R/R cohort, the median OS was 9.7 months with a 1-year OS rate of 32%, and the median OS was not reached when censored for SCT. Two patients had early mortality in the post-SCT period due to infection and graft versus host disease. Patients treated in first relapse had a significantly superior OS than those treated in second or later relapse (median OS not reached vs 5.2 mo; 1-year OS 75% vs 10%, HR 0.24, 95% CI 0.07–0.79, P = 0.04) (Fig. 2). In patients treated with ENA + AZA + VEN, at a median follow up of 11.2 months, median OS and EFS were not reached and 6-mo OS and EFS were 70% and 67%, respectively. Although the sample size is small, R/R AML patients treated with ENA + AZA + VEN exhibited a trend towards better OS than those treated with ENA + AZA (median OS not reached vs 6.0 months,1-year OS 67% vs 20%, HR 0.29,95% CI 0.09–0.97 P = 0.08) (Fig. 3).

### Exploratory outcomes

**Fig. 4:**
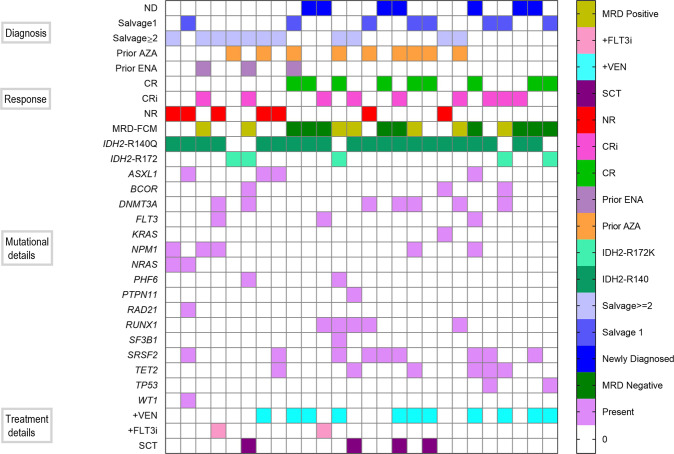
Landscape of entire cohort with genomics and outcomes. Mutational landscape of the whole cohort.

Co-occurring mutational patterns with response and treatment details are shown in Fig. 4. Variant allele frequency (VAF) of *IDH2*^mut^ at the start of treatment was available in 22 patients and ranged from 3% to 50%. The median VAF of *IDH2*^mut^ did not differ greatly between p.R140Q (n = 17) and p.R172K (n = 5) variants (36% and 32%, respectively). Sequential NGS was available for 16 patients (ND = 5, R/R = 11) at the time of response evaluation. Persistent *IDH2*^*mut*^ was detected in all patients (n = 16). Among all responding patients with ND *IDH2*^mut^ AML, the median VAF decrease was greater than 50% (median decrease of 42% to 18%). Among the responding patients with R/R *IDH2*^mut^ AML, the median VAF decreased from 32% to 19%. In R/R *IDH2*^mut^ AML, four evaluable patients with p.R172K variant achieved CR/CRi and had numerically better survival compared to those with p.R140Q variant (11.4 vs 5.2 months, 6-mo OS 75% vs 49%, P = 0.36). While flow MRD negativity was common, no responding patients attained *IDH2* negative status by NGS (sensitivity 1%).

Common co-occurring mutations among the entire cohort involved *SRSF2* (38%), *DNMT3A* (31%), and *RUNX1* (23%) followed by *NPM1*, *ASXL1*, *TET2* (20% each). *KRAS/NRAS* (24%) and *TP53* (8%) mutations were exclusively seen in the R/R group. Patients with ND AML had fewer number of mutations (median = 3, range 1–7) than the R/R group (median = 4, range 1–7) with no difference between responders and non-responders. Molecular subgroups conferring treatment resistance to ENA [22, 23] [*K*RAS/*NRAS* (86%)*, SRSF2* (57%)*, ASXL1* (57%), *DNMT3A, RUNX1*, and *BCOR*(14% each)] were enriched in non-responders. The CRc rate was 64% in patients without *KRAS/NRAS* and *TP53* mutations. In contrast, the CRc rate was only 40% in patients with *KRAS/NRAS* and/or *TP53* mutations.

### Safety

All patients were evaluable for safety. The median number of treatment cycles received was three (range, 0–18) including four in responding and three in non-responding patients. ENA + AZA combination was reasonably well tolerated (Table 3). Thirty- and 60- day mortality rates were 0% each in the ND group and 5% each (n = 1, each) in the R/R group. At the time of data cut off, eight patients (31%) remain on treatment, and reasons for treatment discontinuation include HSCT (*n* = 4, 15%), disease progression (*n* = 4,15%), and death (*n* = 10: disease progression =5, relapse=4, CRi =1).

**Table 3:** Treatment emergent adverse events in newly diagnosed and relapsed/refractory patients with *IDH2* mutant acute myeloid leukemia.

TEAE	Newly diagnosed *n* = 7	Relapsed/refractory *n* = 19
IDH-DS	1 (14)	1 (5)
Febrile neutropenia ≥Gr 3	1 (14)	5 (26)
ALT ≥ Gr 3	0	1(5)
AST ≥ Gr 3	0	1(5)
Total bilirubin ≥Gr 3	2 (29)	7(37)
**GI (any grade)**		
Nausea	0	1 (5)
Vomiting	0	2(11)
Diarrhea	0	4 (21)

*TEAE-* Treatment emergent adverse event, *IDH-DS-* IDH inhibitor induced differentiation syndrome, *AST-* Aspartate aminotransferase, *ALT-* Alanine aminotransferase, *GI-* Gastrointestinal

All results expressed as No. (%) or median [Minimum - maximum], unless specified.

Treatment-emergent adverse events (TEAE) ≤ grade 3 were reported in 22/26 pts (85%). Most frequent grade ≤3 TEAEs include febrile neutropenia (23%), and indirect hyperbilirubinemia (35%). Gastrointestinal AEs of any grade included vomiting (11%) and diarrhea (21%). Tumor lysis syndrome (0%) was not observed in patients treated with ENA + AZA + VEN combination. Most TEAEs were manageable without dose interruption.

IDH-DS was reported in 2 pts (8%) of which one received concurrent gilteritinib for *FLT3/IDH2*^comutated^ AML. In this patient, IDH-DS (leukocytosis, grade 1 creatinine increase) was observed on D18 of treatment and was managed with steroids with normalization of kidney function. Further treatment was continued without dose interruption or modifications. ENA was held in the other patient who developed IDH-DS (fever, pleural effusion, pulmonary infiltrates on imaging) on D31 of treatment and was managed with steroids. No patient died due to IDH-DS.

## Discussion

Treatment options remain limited in patients with AML, particularly those patients not eligible for intensive chemotherapy or in patients with relapsed/refractory disease. The presence of an *IDH2* mutation identifies important treatment options including targeted mutant IDH2i therapy. In ND *IDH2*^mut^ AML, we report a CRc of 100% with 85% CR with ENA + AZA and at a median follow-up of 13.1 months, the median OS was not reached. Notably four patients received ENA + AZA + VEN triplet combination. The outcomes of ENA + AZA are in line with azacitidine and venetoclax (84% CRc, median OS – not reached) combination [24], as well as the outcomes of ENA + AZA in a randomized phase 2 trial with CRc rate 63% and median OS 22 months in patients with ND *IDH2*^mut^ AML [25].

R/R AML is notoriously treatment-resistant as shown by an ORR of 14–28% in a study evaluating three different treatment regimens in patients with primary refractory disease or in first relapse [26]. In our R/R *IDH2*^mut^ AML cohort that mostly included heavily pretreated patients, we report a CRc of 58%, with 28% CR with ENA + AZA combination. Patients in first relapse had numerically higher CRc and a significantly better OS than those in second relapse or later. This significant survival benefit seen among patients in first relapse suggest an earlier therapeutic role for ENA + AZA combination in patients with R/R *IDH2*^mut^ AML. This is consistent with the data from ENA monotherapy, where both response rates and OS decreased in more heavily pretreated patients [22].

The ENA + AZA + VEN “triplet” combination in R/R *IDH2*^mut^ AML was well tolerated and patients with prior HMA or ENA continued either of these agents with VEN. ENA + AZA + VEN triplet combination demonstrated a CRc of 86% even in those with prior exposure to AZA or ENA. The median OS was not reached, and the 1-year OS was 67%. Encouraging outcomes with ENA + AZA + VEN compare favorably to decitabine-10 days and venetoclax combination with CR/CRi rate of 54% and median OS of 14.7 months in R/R *IDH2*^mut^ AML [27].

While responding patients did demonstrate modest reductions in the *IDH2* VAF compared to non-responding patients, “clearance” of the *IDH2* ^mut^ to <1% was not identified in any responding patient at the time of this analysis. This is consistent with prior reports stating response is not dependent on molecular clearance, and in line with data from the ENA + AZA vs AZA alone trial for newly diagnosed older AML, where the median maximum *IDH2* VAF reduction was 81.5% and occurred in responding patients with a median of 6 cycles of the combination [25]. Additionally, given the enrichment of MDS-type mutations in this cohort, it is likely the *IDH2* remains as persistent of a “CHIP” clone in many of these patients, despite clearance of the AML. Whether deeper responses are associated with more durable and prolonged remissions is an important question for future follow up. Additionally, concomitant receptor tyrosine kinase mutations and *TP53* mutations were associated with primary and secondary resistance in the relapsed *IDH2*^mut^ population as has been previously described [22, 23]. While *TET2* mutations are uncommon in the setting of *IDH2* mutations due to biologically overlapping pathogenic mechanisms [28], in our cohort, five (20%) patients harbored *TET2* co-mutations, and all of them were responders. Whether these mutations were found in the same or different leukemic clones in these patients is not known.

ENA + AZA combination was generally well tolerated. The most common non-hematologic toxicity was transient indirect hyperbilirubinemia (35%) that did not warrant treatment interruption, which is a known side effect of enasidenib due to UGT1A1 metabolism. Early mortality rate in ND patients was 0% and was 5% for R/R group, which was reassuring [9, 10]. ENA is known to cause differentiation syndrome (IDH-DS) by virtue of its activity as a differentiating agent. IDH-DS occurred with anticipated frequencies, and was lower with ENA + AZA (8%) than ENA monotherapy (11.7%) [29]. Among the two patients who developed IDH-DS, one patient received concurrent gilteritinib which has also been known to cause differentiation syndrome [30]. Prompt initiation of steroids and supportive measures rapidly mitigated the signs and symptoms of IDH-DS. The safety profile of ENA + AZA combination was comparable to ENA monotherapy [29] and no new TEAEs were observed.

The limitations of our study include a lack of comparator arm and the inherent risk of selection bias. To minimize selection bias, we enrolled consecutive *IDH2*^mut^ AML patients who met the study eligibility criteria, and the eligibility criteria allowed prior therapies (including ENA or HMA or VEN) and/or antecedent hematologic neoplasms and ECOG PS up to 3, to try to replicate a more “real world” patient population.

In summary, ENA + AZA is a safe and effective treatment combination, both for ND elderly pts, as well as in R/R AML, and even those patients having previously received prior HMA or ENA. This pilot evaluation of ENA + AZA in R/R *IDH2*^mut^ AML suggests an earlier therapeutic role of ENA, with improved responses seen in patients treated during their first relapse. Furthermore, the “triplet” of ENA + AZA + VEN appears to be improve outcomes in high risk, heavily pretreated R/R *IDH2*^mut^ AML. Ongoing systematic evaluation of an all oral regimen incorporating decitabine/cedazuridine, enasidenib and venetoclax may confirm the activity of this combination in R/R *IDH2*^mut^ AML. (NCT04774393)
